# Modeling items for text comprehension assessment using confirmatory factor analysis

**DOI:** 10.3389/fpsyg.2022.966347

**Published:** 2022-10-20

**Authors:** Monika Tschense, Sebastian Wallot

**Affiliations:** ^1^Department of Language and Literature, Max Planck Institute for Empirical Aesthetics, Frankfurt, Germany; ^2^Research Group for Research Methods and Evaluation, Institute of Psychology, Leuphana University Lüneburg, Lünebrug, Germany; ^3^Research Group for Neurocognition of Music and Language, Planck Institute for Empirical Aesthetics, Frankfurt, Germany

**Keywords:** reading, text comprehension, reading comprehension, comprehension assessment, discourse representation, mental model

## Abstract

Reading is a complex cognitive task with the ultimate goal of comprehending the written input. For longer, connected text, readers generate a mental representation that serves as its basis. Due to limited cognitive resources, common models of discourse representation assume distinct processing levels, each relying on different processing mechanisms. However, only little research addresses distinct representational levels when text comprehension is assessed, analyzed or modelled. Moreover, current studies that tried to relate process measures of reading (e.g., reading times, eye movements) to comprehension did not consider comprehension as a multi-faceted, but rather a uni-dimensional construct, usually assessed with one-shot items. Thus, the first aim of this paper is to use confirmatory factor analysis (CFA) to test whether comprehension can be modelled as a uni-or multi-dimensional concept. The second aim is to investigate how well widely used one-shot items can be used to capture comprehension. 400 participants read one of three short stories of comparable length, linguistic characteristics, and complexity. Based on the evaluation of three independent raters per story, 16 wh-questions and 60 yes/no-statements were compiled in order to retrieve information at micro and inference level, and 16 main contents were extracted to capture information at the macro level in participants’ summaries. Still, only a fraction of these items showed satisfactory psychometric properties and factor loadings – a blatant result considering the common practice for item selection. For CFA, two models were set up that address text comprehension as either a one-dimensional construct (a uni-factor model with a single comprehension factor), or a three-dimensional construct reflecting the three distinct representational levels (three correlated first-order factors). Across stories and item types, model fit was consistently better for the three-factor model providing evidence for a multi-dimensional construct of text comprehension. Our results provide concrete guidance for the preparation of comprehension measurements in studies investigating the reading process.

## Introduction

As we read, some kind of mental representation of the semantic structure of the text has to be generated, and – as long as reading progresses and new material (i.e., words) is processed – this model has to be expanded and updated constantly (Verhoeven and Perfetti, 2008; O’Brien and Cook, 2015).

As proposed by Kintsch and Van Dijk (1978), there are two levels to describe the semantic representation of a text, a local micro level and a more global macro level. The basic assumption is that every sentence of the text usually conveys at least one meaning (proposition). The micro level then refers to the whole set of propositions of the text, displaying only linear or hierarchical relations. However, the initial set of propositions has to be reduced and further organized in order to establish connections to the topic of discourse, but also to cope with cognitive limitations such as working memory capacity (Palladino et al., 2001; Radvansky and Copeland, 2001; Butterfuss and Kendeou, 2018). This results in a “meaningful whole” (Kintsch and Van Dijk, 1978, p. 366), a cohesive macro level of informational structure.

A third representation level, the so-called situation model or mental model, furthermore incorporates a reader’s world knowledge and provides a scope for their own deductive and interpretive processes (Graesser et al., 1997; Van Den Broek et al., 2005; Sparks and Rapp, 2010). Thus, inferences can emerge that might exceed the literal meaning conveyed by a text (Perrig and Kintsch, 1985; Graesser et al., 1994, 1997). Since this theory considers both, first the construction of an (elaborated) propositional representation, and further the integration of readers’ knowledge to form a final mental representation of a text, it is known as the construction-integration model (Wharton and Kintsch, 1991; Kintsch, 2005, 2018). While many more theories and models of text comprehension have been proposed, there is also a broad consensus that the representational structure described above is at the core of the vast majority of these theories and models (for a comprehensive review see McNamara and Magliano, 2009).

Previous research has found evidence that comprehension processes at each of these different levels are necessary (e.g., Perrig and Kintsch, 1985; Fletcher and Chrysler, 1990; McKoon and Ratcliff, 1992; Graesser et al., 1994; McNamara et al., 1996; Perfetti and Stafura, 2014; Kintsch, 2018; Lindgren, 2019), but there has been little research assessing comprehension at these different levels simultaneously. Moreover, current studies that investigated text comprehension in relation to process measures of reading did not assess and/or analyse comprehension scores according to different processing stages. For example, when factors such as text difficulty or inconsistencies and their effects on process measures of reading were investigated, comprehension was usually assumed but not explicitly tested (e.g., Rayner et al., 2006; for a review: Ferreira and Yang, 2019). Other studies relating the reading process to comprehension tried to assess comprehension by means of multiple-choice questions, but most of the time further information about how these items were compiled and/or which processing level they relate to were missing (e.g., LeVasseur et al., 2006, 2008; Wallot et al., 2014, 2015; O'Brien and Wallot, 2016). But even when different items for different processing levels were used (e.g., Schröder, 2011; Mills et al., 2017; Southwell et al., 2020), this differentiation was ultimately lost for further analyses due to averaging to uni-dimensional comprehension scores.

It should be noted that in none of the studies above pre-tests for item comprehensibility, difficulty or consistency were mentioned. It thus has is to be assumed that one-shot items were used in order to asses reader’s text comprehension, relying heavily on the experimenters’ intuition. With regards to post-hoc quality checks, Schröder (2011) was the only one implementing a comprehension evaluation by three independent raters, and was able to show a moderate level of inter-rater agreement (Fleiss’ *κ* = 0.64). Furthermore, only Mills et al. (2017) included a reliability analysis and assessed the internal consistency of their comprehension items. However, this was a post-hoc analysis, and the resulting values for Cronbach’s α ranged from 0.43 (unacceptable) to 0.86 (good) between texts, indicating high variability in item quality.

Looking at the respective findings, it is striking that in some of the referenced studies process measures of reading, e.g., reading times or eye movements, did relate to text comprehension (LeVasseur et al., 2008; Schröder, 2011; Southwell et al., 2020), but that these effects were lacking in others (LeVasseur et al., 2006; Wallot et al., 2015). Moreover, even when process measures were linked to participants’ comprehension scores, effect sizes varied considerably depending on reading tasks (Wallot et al., 2014), data sets (Mills et al., 2017), or age groups (O'Brien and Wallot, 2016). Among the studies investigating the reading process in terms of self-paced reading, word reading speed generally did not predict comprehension well, often producing null-findings, while auto-correlation properties of the fractal scaling type of reading times fared somewhat better (Wallot et al., 2014, 2015; O'Brien and Wallot, 2016). Among the eye movement studies, the models predicting comprehension successfully did not do so based on the same process features (Wallot et al., 2015; Southwell et al., 2020). This state of affairs might be a question of how the reading process was modeled (i.e., which features of the reading process are of importance, and in which combination). However, the problem might also be the result of how the studies referenced above handled the measurement of reading comprehension.

All the studies mentioned above that tried to relate the reading process to comprehension seemed to have worked with one-shot items assessing comprehension through items with little to no systematic pretesting, and without establishing psychometric properties of these items before application. Moreover, they seemed to implicitly assume that comprehension is a uni-dimensional concept, with comprehension being mainly high or low (or present or absent) by averaging all items, or even using Cronbach’s α as an indicator of reliability. However, to the degree that different levels at which comprehension can take place are distinguishable, a uni-dimensional concept might be misleading. The criticism raised here also applies to our own past work, which has followed the same practice and made the same assumptions (Wallot et al., 2014, 2015; O'Brien and Wallot, 2016). Accordingly, we are curious to find out, how good this practice of generating one-shot items can be in terms of producing reliable measures of comprehension, and in how far the assumption of uni-dimensionality is warranted in order to potentially improve future work.

Hence, the aim of the current study is to investigate how good the measurement properties of sets of one-shot comprehension questions are. Moreover, we aim to test whether and how items for comprehension assessment that target different levels of discourse structure (micro vs. macro vs. inference level) jointly contribute to text comprehension. For this purpose, we intend to deduce whether text comprehension can be measured and modelled as a uni-dimensional or multi-dimensional construct by means of confirmatory factor analysis (CFA). Additionally, as exploratory questions, we will investigate the relation between participants’ text comprehension, their liking and interest ratings, as well as text reading times.

## Materials and methods

The methods described below were approved by the Ethics Council of the Max Planck Society. Before inspection of any data, the study was preregistered *via* Open Science Framework (OSF^1^).

### Participants

In total, 400 participants were recruited by distributing leaflets in local pedestrian zones, cafés, libraries, book stores and cinemas, placing advertisements at the institute’s homepage and Facebook, as well as contacting participants *via* email using an in-house database and open email lists. At the end of the survey, participants could decide to join a lottery to win a book voucher of 10 € with odds of one in five. All participants were native speakers of German and at least 18 years old.

Two participants were excluded due to missing data of comprehension items and summary. Another 15 participants’ data was excluded based on text reading times of less than 5 min or more than 40 min. Thus, the final sample consisted of 383 participants (302 females, 79 males, 2 others) with an age range between 19 and 91 years (*M* = 47.05, *SD* = 16.29). A majority of 69.45% of the participants stated holding a higher education degree. With regard to reading habits, participants reported to spend an average of 20.17 h per week (*SD* = 14.31) reading, for instance, books, newspaper articles, and blog posts. Participants were randomly assigned to one of three short stories, see Table 1 for distribution of demographic variables per text.

**Table 1:** Participant demographics.

Short story	*N*	Sex			Age (years)			Reading per week (hours)		Educational level			
		Female	Male	Other	Range	*M*	*SD*	*M*	*SD*	Higher edu. entrance	Vocational qualification	Higher education	Other
1	117	93	24	0	[19, 77]	47.24	16.98	19.16	12.89	22	11	83	1
2	126	98	27	1	[19, 77]	46.42	14.32	20.38	12.41	13	16	91	6
3	140	111	28	1	[19, 91]	47.46	17.41	20.82	16.85	32	13	92	3
Overall	383	302	79	2	[19, 91]	47.05	16.29	20.17	14.31	67	40	266	10

Reading per week refers to the self-reported number of hours that participants approximately read per week (including books, newspaper articles, blog posts, etc.).

### Materials

#### Texts

To allow for some generalization of the results across different texts, three short stories with different topics, but comparable complexity of content and pace of narration were selected. Short story 1 (“Brief an Juliane” [Letter to Juliane] by Hosse, 2009) describes the circumstances and challenges of growing up after World War II in an autobiographical manner (first-person narration). In contrast, short story 2 (“Die verborgene Seite der Medaille” [The hidden side of the coin] by Scavezzon, 2010) is a more typical short story with a third-person selective narrator and a plot twist towards its open end. Here, fact and fiction blend into one elaborate metaphor about the life of the main character, a veteran pilot that was involved in the bombing of Hiroshima. Short story 3 (“Der Doppelgänger” [The doppelganger] by Strauß, 2017) is a third-person omniscient narrative featuring a woman with Capgras syndrome, a psychological disorder leading her to the delusion that her husband has been replaced by an identical-looking impostor.

If necessary, the stories were adapted to current German spelling rules. Where possible, direct speech was either omitted or paraphrased. The texts were then shortened to a length of roughly 3,000 words to achieve a reading time of approximately 10–15 min (Brysbaert, 2019). The short stories were matched for number of words per sentence and mean length of words based on both, number of graphemes and number of syllables per word. Moreover, average logarithmic word frequencies obtained from dlexDB (Heister et al., 2011) were similar for all texts. See Table 2 for more information regarding text characteristics.

**Table 2:** Key characteristics per text.

Short story	Words	Sentences	Words per sentence	Graphemes per word	Syllables per word	Type frequency		Type frequency DC		Annotated type frequency	
						Absolute	log10	Absolute	log10	Absolute	log10
1	3,123	260	12.01	5.31 (2.99)	1.75 (0.96)	406,824.70 (785,206.60)	4.40 (1.25)	503,086.36 (914,730.01)	4.50 (1.54)	343,320.31 (704,039.84)	4.20 (1.57)
2	2,967	244	12.16	5.02 (2.72)	1.69 (1.02)	371,672.56 (695,293.86)	4.56 (1.32)	445,139.65 (78,6186.05)	4.66 (1.33)	318,950.96 (635,276.25)	4.38 (1.37)
3	3,113	262	11.88	5.29 (2.92)	1.77 (0.98)	398,567.54 (749,976.33)	4.47 (1.44)	505,960.92 (961,725.28)	4.57 (1.45)	337,254.16 (673,702.76)	4.30 (1.47)

Words and sentences refer to the number of words and number of sentences per story, all other values are averaged per story; standard deviations are given in brackets.

#### Comprehension items

To assess text comprehension as thoroughly as possible, different types of comprehension tasks were used. For each text, 60 yes/no-statements were generated, 40 of these aimed at micro-level content, the remaining 20 at inference-level content. Items assessing micro level comprehension related to information encoded at the sentence-level. Items assessing inferences did not have an explicit reference in the text as they exceed its literal meaning and integrate the reader’s world knowledge. Here is an example:

Original text:

“Lore und ich verdienten uns unser Taschengeld dann beim Großbauern beim Erbsenpflücken, was damals noch per Hand gemacht wurde. Um sechs Uhr in der Frühe traf man sich und wurde zum Feld gekarrt. Zuweilen brannte die Sonne erbarmungslos, aber wir hatten ein Ziel. Wenn man fleißig war, hatte man am frühen Nachmittag einen Zentner, also fünfzig Kilogramm. Das war mühsam, denn Erbsen sind leicht. Man bekam dafür drei D-Mark, ein kostbarer Schatz, den man hütete.”

*[Lore and I then earned our pocket money by picking peas at a large farm, which was still done by hand at that time. We met at six in the mornings and were taken to the field. Sometimes the sun burned mercilessly, but we had a goal. If you were diligent, you got fifty kilograms by early afternoon. It was exhausting, because peas are light. In return we received three German marks, a precious treasure that we guarded.]*

Item for micro information:

Die Protagonistin half beim Erbsenpflücken, um sich Taschengeld zu verdienen.

*The main character helped picking peas to earn some pocket money.*

Item for inferred information:

Die Protagonistin musste schon früh lernen, hart für ihr Geld zu arbeiten.

*The main character had to learn early on to work hard for her money.*

Yes/no-statements provide a widely used and, with regards to procedure and analysis, fast and easy tool to evaluate text comprehension. However, in the absence of prior knowledge about such items, there is a risk of comparably high probability of guessing and the possibility that a certain context or wording may simplify giving the right answer. Therefore, 16 wh-questions with open input fields were compiled for each text, 10 of which for testing comprehension at micro level, the remaining six at inference level.

For both tasks, a larger pool of items was initially prepared with items either referring to a specific part of the story or relating to the overall plot. For yes/no-statements this initial item compilation consisted of 120 items per text, for wh-question an initial pool of 40 items was initially generated Supplementary Material 1. Subsequently, these items were independently judged by three raters. Finally, the best-rated 60 yes/no-statements and 16 wh-questions that were evenly distributed throughout the whole text were selected for data acquisition.

In order to examine text comprehension at macro level, three raters summarized the main contents of each story. Ideas that appeared in all three summaries were maintained; ideas that were mentioned in only two of the summaries were first discussed and subsequently either discarded or maintained. This resulted in 16 main ideas per text which were later on used to evaluate participants’ summaries – i.e., counting the presence or absence of these ideas in each summary.

### Procedure

An online study was set up using the platform SoSci Survey.^2^ The study could be accessed from mid-December 2019 until mid-March 2020. At the beginning of the study, participants were informed about the aims and specific contents of the study, as well as data protection rules. Subsequently, they were asked for some socio-demographic information. Participants were then randomly assigned to one of the three short stories. They were instructed to read the assigned text in a natural manner, if possible, in quiet surroundings and without interruptions. The text was presented as a whole and participants could freely scroll up and down to go back or forth. The text was formatted in HTML with Arial font in size 3. Paragraphs were visually indicated with larger white space between lines. During the experiment, there was no set time limit for reading. On average, participants needed 12.97 min (*SD* = 4.69) to read a text.

After reading the short story, participants were required to write a brief summary reflecting the main contents of the short story. Subsequently, participants first answered the wh-questions followed by the yes/no-statements. All wh-questions were presented in one list but in randomized order. The sequence in which yes/no-statements were displayed was also randomized, and items were distributed across three pages of the survey. Finally, participants were asked to fill out a short questionnaire assessing their reading experience in terms of interest, liking, suspense, urgency, vividness, cognitive challenge, readerly involvement, rhythm, and intensity. To this end, participants were asked to rate how strongly they agree with a presented statement on a seven-point scale ranging from 0 (“not at all”) to 6 (“extremely”). For the purpose of this study, we were only interested in participants’ global interest (“How interested are you in the text?”) and liking (“How much do you like the text?” “How gladly would you like to read similar texts?,” “How strongly would you recommend the text to a friend?”).

### Item selection

Participants’ answers to the wh-qhestions were assessed as true (1) or false (0). Furthermore, the written summaries were evaluated regarding the presence (1) or absence (0) of the 16 main ideas, thus, each summary could have received a maximum of 16 points. For this purpose, two raters familiarized themselves again with the text (i.e., reading the short story and reviewing its main ideas), and subsequently discussed and rated eight randomly drawn summaries together. The raters assessed another two summaries individually and then discussed their evaluations until they agreed upon a final assessment. This training was implemented to ensure best possible inter-rater reliability and took about 1.5 h per short story. Afterwards, both raters individually assessed all summaries corresponding to the respective short story (approximately 5.5 h per rater and text). The order of the summaries was randomized. Indeed, good inter-rater agreement was achieved as indicated by Krippendorf’s α of 0.926 for short story 1, 0.936 for short story 2, and 0.902 for short story 3. Finally, discrepant evaluations were discussed until the raters agreed upon a final rating (roughly 1 h per text).

To filter out items with bad psychometric properties before computing any model, an item analysis was performed. As a first step, individual distributions of the items were inspected. Items that showed an accuracy rate of less than 5% or more than 95% were excluded from further analysis. Subsequently, joint distributions were observed by computing the phi coefficient (*r_ϕ_*) for each pair of items. Since the different types of comprehension items are assumed to evaluate a different level of text comprehension, items of the same type are supposed to correlate with each other while items of different types should be not at all or less strongly correlated. Hence, items were successively excluded until items within a type reached an average *r_ϕ_* between 0.1 and 0.9, and items between types did not exceed an average *r_ϕ_* of 0.25.

With the remaining items a CFA was carried out using the R package *lavaan* (Rosseel, 2012). If the analysis did not converge, additional items were discarded based on their loadings, starting with the item with the lowest loading. When the analysis converged, standardized estimates were assessed and items with values of less than 0.2 and greater than 0.9 were removed.

Following the steps of the item analysis described above, at least three items for each item type could be retained per short story. An overview of the items can be found in Supplementary Material 2.

## Results

The average reading time over all texts was 12.97 min (*SD*  = 4.69), 15.08 min (*SD*  = 4.87) for short story 1, 11.33 min (*SD*  = 4.14) for short story 2, and 12.68 min (*SD*  = 4.36) for short story 3. Participants’ liking and interest ratings were in the medium range with an average score of 3.48 (*SD*  = 1.62) respectively 3.68 (*SD*  = 1.54) across all texts. For short story 1, ratings yielded an average of 3.51 (*SD*  = 1.64) for likability and 4.02 (*SD*  = 1.56) for interest. Short story 2 scored a mean likability rating of 3.68 (*SD*  = 1.61) and a mean interest rating of 3.60 (*SD*  = 1.65). For short story 3, mean likability was 3.27 (*SD*  = 1.60) and mean interest was 3.46 (*SD* = 1.36). Regarding the comprehension items, participants average accuracy rates were 85.25% for yes/no-statements (*SD*  = 16.93; short story 1: *M* = 88.69%, *SD*  = 13.89; short story 2: *M* = 82.88%, *SD* = 17.20; short story 3: *M* = 84.18%, *SD*  = 19.02), 59.03% for wh-questions (*SD*  = 22.60; short story 1: *M* = 61.43%, *SD*  = 19.37; short story 2: *M* = 54.71%, *SD*  = 23.02; short story 3: *M* = 60.95%, *SD*  = 25.79), and 53.87% for the main contents of the summaries (*SD*  = 29.38; short story 1: *M* = 41.35%, *SD*  = 28.03; short story 2: *M* = 66.82%, *SD*  = 27.22; short story 3: *M* = 53.46%, *SD*  = 28.85). Accuracy rates per item are provided in Supplementary Material 2.

### Comparing text comprehension models (CFA)

**Figure 1:**
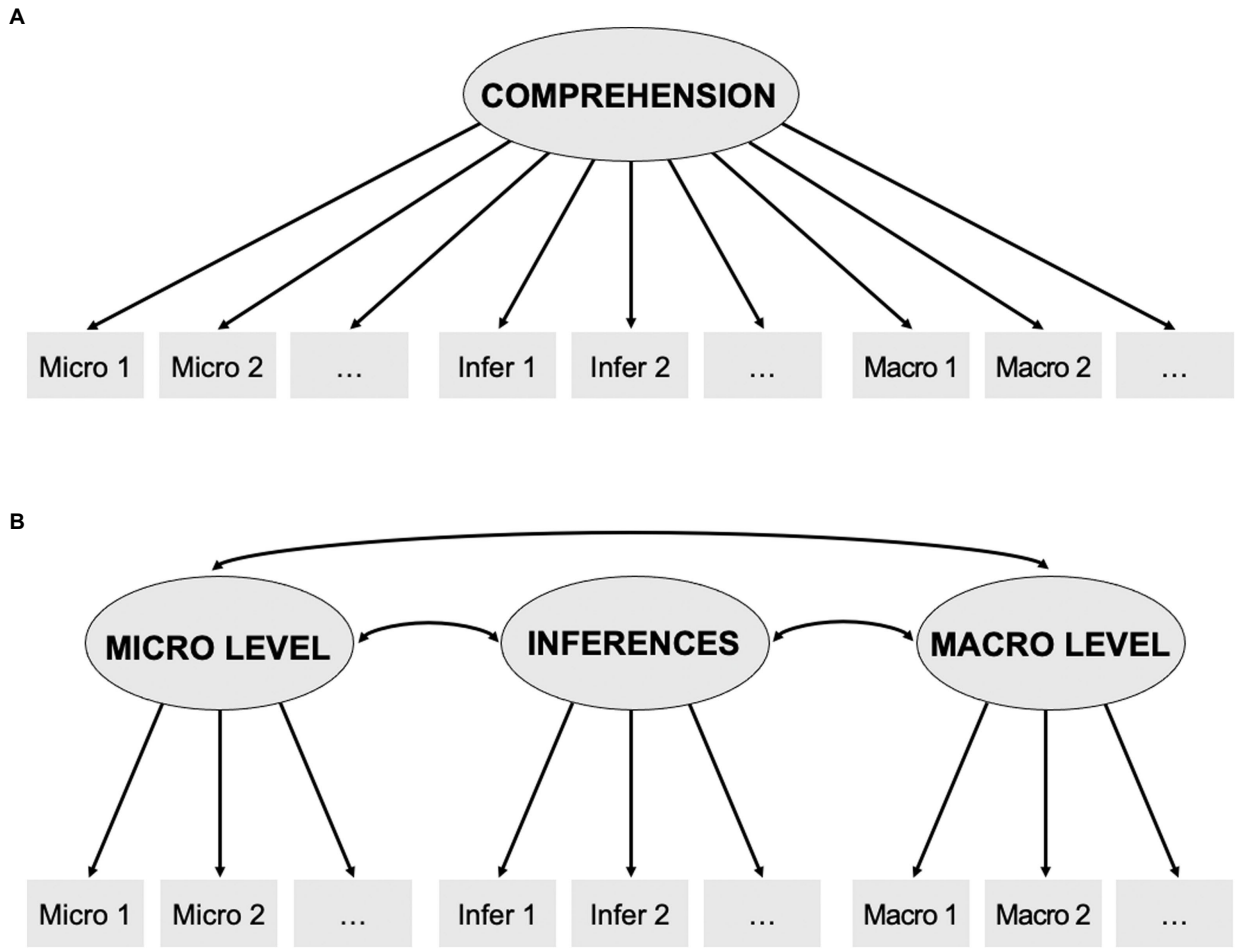
Models for confirmatory factor analysis (CFA). **A:** Uni-factor model of comprehension across three different items types (micro, macro, and inference level). **B:** Model of three correlated factors, assigning each item type its own latent variable.

For each of the short stories, two different models were set up that reflect text comprehension as (A) one-dimensional construct implemented as uni-factor model with a single comprehension factor, or as (B) multi-dimensional construct capturing all levels of text comprehension (micro level, macro level, inferences) designed as a model containing three correlated first-order factors. All models were conducted separately for wh-questions and yes/zo-statements. The specified models are shown in Figure 1. While we first planned to compute a third model based on the same multi-dimensional construct as in (B), extended by a second-order factor reflecting higher-level, general comprehension, this could not be realized due to converging errors.

Table 3 contains information about the goodness-of-fit indicators for each of the models described above. Both, unstandardized and standardized estimates are shown in Supplementary Material 3. When looking at yes/no-statements, model fit across all short stories is better for the three-factor model as compared to the uni-factor model. Turning towards the wh-questions, the same pattern emerges: Across all short stories, better model fit is indicated for the three-factor model than for the uni-factor model. When comparing the two types of comprehension tasks, some fit indices show even better model fit for wh-questions compared to yes/no-statements. Again, this pattern can be seen across all three short stories. In sum, the assumption that comprehension is a one-dimensional concept did not receive support from our model analysis. Note, that none of the models did converge when set up with the whole set of items; neither did the higher-order factor model.

**Table 3:** Model fit per text.

Short story	Comprehension task	Model	ChiSQ				CFI	TLI	RMSEA			SRMR
			Value	df	ChiSQ / df	*p*			Value	90% CI	*p*	
1	Yes / no statements	A: uni-factor model	150.35	119	1.26	0.027	0.90	0.89	0.05	[0.017, 0.070]	0.549	0.21
		B: three-factor model	109.11	116	0.94	0.662	1.00	1.03	0.00	[0.000, 0.040]	0.989	0.18
	Wh-questions	A: uni-factor model	79.55	77	1.03	0.399	0.99	0.99	0.02	[0.000, 0.056]	0.905	0.15
		B: three-factor model	53.39	74	0.72	0.966	1.00	1.11	0.00	[0.000,0.000]	0.999	0.13
2	Yes / no statements	A: uni-factor model	166.73	152	1.10	0.196	0.91	0.90	0.03	[0.000, 0.051]	0.936	0.18
		B: three-factor model	103.46	149	0.69	0.998	1.00	1.30	0.00	[0.000, 0.000]	1.000	0.14
	Wh-questions	A: uni-factor model	116.63	90	1.30	0.031	0.78	0.74	0.05	[0.016,0.072]	0.516	0.15
		B: three-factor model	76.17	87	0.88	0.790	1.00	1.11	0.00	[0.000, 0.034]	0.994	0.12
3	Yes / no statements	A: uni-factor model	223.04	170	1.31	0.004	0.78	0.75	0.05	[0.028,0.064]	0.587	0.17
		B: three-factor model	153.68	167	0.92	0.762	1.00	1.06	0.00	[0.000, 0.028]	1.000	0.14
	Wh-questions	A: uni-factor model	69.89	77	0.91	0.705	1.00	1.06	0.00	[0.000, 0.038]	0.990	0.13
		B: three-factor model	50.25	74	0.68	0.984	1.00	1.18	0.00	[0.000, 0.000]	1.000	0.11

CFI, comparative fit index; TLI, Tucker-Lewis index; RMSEA, root mean square error of approximation; SRMR, standardized root mean squared residual.

### Relation between comprehension, reading times, global interest and liking

In order to shed light on the relation between participants’ comprehension scores, their ratings for global interest and liking of the text, as well as their reading times, Pearson’s product–moment-correlation was computed for each pair of variables across short stories. To this end, reading time was logarithmized to adjust for normality, comprehension scores for the different discourse levels (micro vs. macro vs. inference level) were divided by their respective number of items, and an overall comprehension sum score was derived in the same manner, before all variables were z-transformed per short story. Results are shown in Table 4 for wh-questions, and in Table 5 for yes/no-statements.

**Table 4:** Correlation matrix for wh-questions (selected items).

		Micro	Macro	Inference	Interest	Liking	Log reading time
Story 1	Micro	–	0.13	0.26^**^	−0.04	0.04	0.12
	Macro	0.13	–	0.23^*^	−0.06	0.01	0.08
	Inference	0.26^**^	0.23^*^	–	0.27^**^	0.26^**^	0.15
	Interest	−0.04	−0.06	0.27^**^	–	0.74^***^	0.09
	Liking	0.04	0.01	0.26^**^	0.74^***^	–	0.11
	Log reading time	0.12	0.08	0.15	0.09	0.11	–
Story 2	Micro	–	0.06	0.28^**^	0.09	0.09	0.13
	Macro	0.06	–	0.10	0.03	0.02	−0.09
	Inference	0.28^**^	0.10	–	−0.03	0.01	0.14
	Interest	0.09	0.03	−0.03	–	0.71^***^	0.03
	Liking	0.09	0.02	0.01	0.71^***^	–	0.02
	Log reading time	0.13	−0.09	0.14	0.03	0.02	–
Story 3	Micro	–	0.11	0.18^*^	0.01	−0.02	0.11
	Macro	0.11	–	0.04	0.04	0.10	0.09
	Inference	0.18^*^	0.04	–	0.12	0.23^**^	0.14
	Interest	0.01	0.04	0.12	–	0.68^***^	0.01
	Liking	−0.02	0.10	0.23^**^	0.68^***^	–	0.03
	Log reading time	0.11	0.09	0.14	0.01	0.03	–
Overall	Micro	–	0.10	0.24^***^	0.02	0.03	0.12^*^
	Macro	0.10	–	0.12^*^	0.01	0.05	0.03
	Inference	0.24^***^	0.12^*^	–	0.12^*^	0.17^**^	0.14^**^
	Interest	0.02	0.01	0.12^*^	–	0.71^***^	0.04
	Liking	0.03	0.05	0.17^**^	0.71^***^	–	0.05
	Log reading time	0.12^*^	0.03	0.14^**^	0.04	0.05	–

Pearson’s *r* correlation coefficients. ^*^p < 0.05; ^**^p < 0.01; ^***^p < 0.001.

**Table 5:** Correlation matrix for yes/no statements (selected items).

		Micro	Macro	Inference	Interest	Liking	Log reading time
Story 1	Micro	–	0.08	0.24^**^	0.03	0.14	0.14
	Macro	0.08	–	0.02	−0.03	0.00	0.02
	Inference	0.24^**^	0.02	–	−0.03	−0.02	0.01
	Interest	0.03	−0.03	−0.03	–	0.74^***^	0.09
	Liking	0.14	0.00	−0.02	0.74^***^	–	0.11
	Log reading time	0.14	0.02	0.01	0.09	0.11	–
Story 2	Micro	–	0.05	−0.04	0.11	0.10	0.18^*^
	Macro	0.05	–	−0.08	0.04	0.04	−0.07
	Inference	−0.04	−0.08	–	0.07	0.06	0.07
	Interest	0.11	0.04	0.07	–	0.71^***^	0.03
	Liking	0.10	0.04	0.06	0.71^***^	–	0.02
	Log reading time	0.18^*^	−0.07	0.07	0.03	0.02	–
Story 3	Micro	–	−0.03	0.11	−0.05	−0.02	0.12
	Macro	−0.03	–	0.07	0.03	0.08	0.12
	Inference	0.11	0.07	–	−0.10	−0.02	0.17^*^
	Interest	−0.05	0.03	−0.10	–	0.68^***^	0.01
	Liking	−0.02	0.08	−0.02	0.68^***^	–	0.03
	Log reading time	0.12	0.12	0.17^*^	0.01	0.03	–
Overall	Micro	–	0.03	0.10^*^	0.03	0.07	0.15^**^
	Macro	0.03	–	0.01	0.02	0.04	0.03
	Inference	0.10^*^	0.01	–	−0.02	0.01	0.09
	Interest	0.03	0.02	−0.02	–	0.71^***^	0.04
	Liking	0.07	0.04	0.01	0.71^***^	–	0.05
	Log Reading Time	0.15^**^	0.03	0.09	0.04	0.05	–

Pearson’s *r* correlation coefficients. ^*^p < 0.05; ^**^p < 0.01; ^***^p < 0.001.

As is evident in the correlation matrix, the different levels of text processing only show weak correlations among each other. This is true for both, wh-questions and yes/no-statements. As could be expected, participants’ global interest and liking of a short story are strongly correlated. However, a better reading experience does not relate to better comprehension of a text in a meaningful way. Furthermore, there is no strong evidence for a correlation between text comprehension and participants’ reading times.

The pre-selection of comprehension items as described above descriptively leads to somewhat better discriminatory power between the three levels of text processing: There is a slight decrease in correlation coefficients for the selected items as compared to the whole item set. However, the overall relations between the investigated variables do otherwise remain the same. Correlation results for the whole item set across texts are displayed in Supplementary Material 4.

## Discussion

The current study had two aims: First, we wanted to simultaneously model the three processing levels of comprehension (micro, macro and inference level). Particularly, we were interested in comparing a uni-factor model (i.e., that comprehension behaves the same across all of these three levels) with a model that assigns each of these levels their own factor. Second, we wanted to test the quality of different comprehension items in terms of capturing text comprehension after reading. This second point relates to the common practices of comprehension assessment, especially as applied in studies investigating the relation between process measures of reading and text comprehension. Here, researchers often seem to work with one-shot items of unknown psychometric quality, and to implicitly assume that comprehension is effectively a one-dimensional construct.

Our results indicated that a three-factor model of text comprehension fits our data significantly better than a uni-factor model. This was true for all three short stories and regardless of item type. Consequently, we provided evidence that comprehension should indeed be considered a three-dimensional construct. At the same time, our results showed that all three processing levels were correlated. This suggests three related, yet distinct levels of comprehension influencing one another. Thus, our analysis yields complementary evidence to studies investigating specific aspects of these processing levels separately. Accordingly, our results are in line with the assumption of three representational levels of discourse comprehension (micro, macro and inference level; *cf.* Kintsch and Van Dijk, 1978), also when these three levels are investigated simultaneously. In line with the theory, the results suggested a model with correlated factors, indicating that these levels are separate, but interdependent (*cf*. Perrig and Kintsch, 1985; Fletcher and Chrysler, 1990; McNamara et al., 1996; Perfetti and Stafura, 2014; Kintsch, 2018).

However, we would like to point out three aspects of our analysis that were somewhat striking. First, the standardized root mean squared residual (SRMR) values were quite high (≥0.11) for all models that converged, even though other fit indices were in the commonly expected range. Such larger SRMR values were reported before in the case of relatively small sample sizes of 200 or less due to higher degrees of uncertainty or variability that come along with smaller samples (*cf*. Taasoobshirazi and Wang, 2016). Second, when the whole initial item set was used in the comprehension models, none of the models converged. Thus, a comparison between the whole item pool and selected items was not possible indicating that items of poor and/or heterogenous quality are difficult to lump together into a single comprehension score. Third, it should be noted again that a higher-order factor model of text comprehension did not converge, indicating model misspecification. Even though this means we have no model fit indices to compare, it suggests that this is not an appropriate way to model the comprehension data.

As laid out in the introduction, it is currently common practice to assess comprehension in terms of one-shot items which are largely based on the experimenter’s intuition for item selection than on theory, pre-tests or post-hoc quality control. As the current study showed, it is of importance to control comprehension items better, even if it requires quite some extra effort. The immense drop-out rate suggests that neither working with independent raters nor basing items on a theory by itself is enough to guarantee high item quality. Pre-testing items and/or reducing items post-hoc in a step-wise manner should be considered when planning further studies that aim to investigate text comprehension processes. Without investing some time and effort on item selection, there is a high risk that comprehension is not assessed in a valid manner and thus cannot be used in order to predict other measures of the reading process.

As we have summarized above, when we compared different studies relating reading process measures to comprehension, very different models emerge, and similar predictors behave differently across these studies (LeVasseur et al., 2006, 2008; Schröder, 2011; Wallot et al., 2014, 2015; O'Brien and Wallot, 2016; Mills et al., 2017; Southwell et al., 2020). This might be due to differences inherent in the specific reading situations (Wallot, 2016), but it might also be a function of varying quality of the comprehension assessment. Please note, that the current study was not a laboratory study, and accordingly, we had little control or information about the time course of reading behavior or the specific reading situation. Even though stricter experimental control is desirable in future work along these lines, this does not invalidate the main conclusion that can be drawn from our results: In order to draw reliable inferences about reading process measures that are related to reading comprehension, reliability and validity of comprehension measures is a necessary prerequisite. If the quality of comprehension measurements is unknown, however, it becomes difficult to trace back why a particular model of reading process measures was successful or failed in predicting reading comprehension as outcome.

## Data availability statement

The dataset for this study is available in the online repository Open Science Framework (OSF): https://osf.io/b2zem/.

## Ethics statement

The studies involving human participants were reviewed and approved by Ethics Council of the Max Planck Society. The patients/participants provided their written informed consent to participate in this study.

## Author contributions

MT designed the experiment and collected and analyzed the data. MT and SW jointly developed the research idea, contributed to the conceptualization of the study, interpreted the results, and wrote the manuscript. All authors contributed to the article and approved the submitted version.

## Funding

The study was funded by the Deutsche Forschungsgemeinschaft (DFG, German Research Foundation) by grants to SW (project numbers 397523278 and 442405852).

## Conflict of interest

The authors declare that the research was conducted in the absence of any commercial or financial relationships that could be construed as a potential conflict of interest.

## Publisher’s note

All claims expressed in this article are solely those of the authors and do not necessarily represent those of their affiliated organizations, or those of the publisher, the editors and the reviewers. Any product that may be evaluated in this article, or claim that may be made by its manufacturer, is not guaranteed or endorsed by the publisher.

## Footnotes

^1^ https://osf.io/2u43j

^2^ https://www.soscisurvey.de
